# Structural and Dynamical Basis of VP35-RBD Inhibition by Marine Fungi Compounds to Combat Marburg Virus Infection

**DOI:** 10.3390/md22010034

**Published:** 2024-01-03

**Authors:** Abdullah S. Alawam, Hadil Sultan Alawam, Mohammed Merae Alshahrani, Maher S. Alwethaynani, Lina M. Alneghery, Mubarak A. Alamri

**Affiliations:** 1 Department of Biology, College of Science, Imam Mohammad Ibn Saud Islamic University (IMSIU), Riyadh 11623, Saudi Arabia; lmalneghery@imamu.edu.sa; 2 College of Medicine, Imam Mohammad Ibn Saud Islamic University (IMSIU), Riyadh 11623, Saudi Arabia; 3 Department of Clinical Laboratory Sciences, Faculty of Applied Medical Sciences, Najran University, Najran 61441, Saudi Arabia; mmalshahrani@nu.edu.sa; 4 Department of Clinical Laboratory Science, College of Applied Medical Sciences, Shaqra University, Al-Quwayiyah 19257, Saudi Arabia; 5 Department of Pharmaceutical Chemistry, College of Pharmacy, Prince Sattam Bin Abdulaziz University, Al-Kharj 11942, Saudi Arabia

**Keywords:** Marburg virus, marine fungi, VP35, RNA binding domain

## Abstract

The Marburg virus (MBV), a deadly pathogen, poses a serious threat to world health due to the lack of effective treatments, calling for an immediate search for targeted and efficient treatments. In this study, we focused on compounds originating from marine fungi in order to identify possible inhibitory compounds against the Marburg virus (MBV) VP35-RNA binding domain (VP35-RBD) using a computational approach. We started with a virtual screening procedure using the Lipinski filter as a guide. Based on their docking scores, 42 potential candidates were found. Four of these compounds—CMNPD17596, CMNPD22144, CMNPD25994, and CMNPD17598—as well as myricetin, the control compound, were chosen for re-docking analysis. Re-docking revealed that these particular compounds had a higher affinity for MBV VP35-RBD in comparison to the control. Analyzing the chemical interactions revealed unique binding properties for every compound, identified by a range of Pi–cation interactions and hydrogen bond types. We were able to learn more about the dynamic behaviors and stability of the protein–ligand complexes through a 200-nanosecond molecular dynamics simulation, as demonstrated by the compounds’ consistent RMSD and RMSF values. The multidimensional nature of the data was clarified by the application of principal component analysis, which suggested stable conformations in the complexes with little modification. Further insight into the energy profiles and stability states of these complexes was also obtained by an examination of the free energy landscape. Our findings underscore the effectiveness of computational strategies in identifying and analyzing potential inhibitors for MBV VP35-RBD, offering promising paths for further experimental investigations and possible therapeutic development against the MBV.

## 1. Introduction

The emergence of the Marburg virus (MBV), belonging to the Filoviridae family, has raised serious public health concerns due to its propensity for causing severe hemorrhagic fevers with high mortality rates in humans [1,2]. In 1967, this RNA virus was identified for the first time in Belgrade, Serbia, and Marburg and Frankfurt, Germany [3]. Since then, it has been linked to other outbreaks in Africa, each characterized by distressingly high fatality rates. The fatal nature of the MBV can be attributed to its intricate interactions with the biological processes of its hosts [4,5].

Central to the virulence of the MBV is its molecular arrangement, especially its array of structural and non-structural proteins that are crucial to the virus’s life cycle [6]. The VP35 protein is mainly known for its dual role in both the viral replication process and the host’s immune system evasion. VP35 binds to double-stranded RNA as an interferon antagonist, preventing the activation of vital host immunological defenses against viral infections [7]. The severe clinical symptoms of MBV infections are caused by the suppression of the immune response, which allows for unchecked viral reproduction [8].

The involvement of VP35 extends to viral RNA synthesis as it collaborates with the RNA polymerase complex. This role highlights its potential as a promising target for MBV therapies [9]. However, the development of effective inhibitors for the VP35-RNA binding domain (VP35-RBD) is challenging, given the complexities of isolating compounds that can specifically target this protein [10]. Currently, the management of MBV infections is primarily supportive, addressing symptoms and complications, with a pressing need for effective therapeutic measures [11]. In recent years, there has been growing interest in the development of highly active inhibitors against MBV [12,13,14]. These inhibitors target various proteins involved in the viral replication cycle, with the goal of blocking viral entry, replication, or assembly. Several promising candidates have emerged from these efforts, and some have shown efficacy in animal models. Several drugs including Favipiravir (L protein) and GS-5734 (VP40 protein) have shown promising inhibitory results against MBV, and these drugs are currently under clinical trials [13,14]. Focusing on VP35 as a therapeutic target is promising, yet it demands an intricate understanding of the protein’s structural and functional dynamics [15].

Although recent reports have highlighted MBV VP35 inhibitors like Semicochliodinol B, Stigmasterol, and Estradiol Benzoate, there is a desperate need for further research in this area [16,17,18]. The complexity and variability of the MBV suggest that a wider array of inhibitors from different sources could be beneficial in developing more effective treatment strategies. Exploring new molecules is important to find more effective and specific inhibitors for targeting MBV VP35, beyond the currently known inhibitors.

The exploration of marine fungi for potential antiviral compounds opens new avenues. These organisms, residing in diverse marine ecosystems, have adapted unique biochemical pathways, yielding a plethora of secondary metabolites with intricate structures and varied biological properties. Marine-derived compounds have historically been a fertile ground for pharmacological discoveries, including several efficacious drugs [19,20].

The vast chemical diversity of marine fungi presents an untapped potential in the quest for novel antiviral agents, especially those targeting the Marburg virus’s VP35 protein. Their distinct biochemical properties, compared to terrestrial counterparts, position them as prime candidates in the search for new therapeutic agents [21].

The MBV represents a formidable challenge in virology research, particularly due to the difficulties associated with treating its infections as there are no effective treatments available for this disease. This gap in treatment options underscores the urgent need for research in this area.

Our work directly addresses this critical issue by focusing on the identification of VP35-targeted antiviral molecules from the marine fungi compound library to combat the MBV. We utilized a comprehensive approach combining virtual screening, adherence to Lipinski’s rule of five, re-docking, molecular dynamics simulations, and principal component analysis for free energy landscape evaluation. This methodology allowed for efficient and extensive screening of marine fungi compounds, focusing on those with potential efficacy against MBV VP35-RBD.

## 2. Results

### 2.1. Virtual Screening and Re-Docking

In our quest to identify novel inhibitors of the Marburg virus VP35 protein, we embarked on a computational drug discovery journey. Utilizing the MtiOpenScreen web server [22], we conducted a virtual screening of a Marine Fungi Compounds library, applying the Lipinski filter to ensure the selection of drug-like molecules. This initial screening resulted in the identification of 42 compounds with promising docking scores, ranging from −5.00 to −6.22 kcal/mol (Table S1).

Myricetin, a well-known inhibitor of the MBV VP35 protein, was taken as a reference molecule for this study [23]. Myricetin docking into the MBV VP35-RBD binding site resulted in a docking score of −4.53 kcal/mol. From this pool, we selected the top four compounds (CMNPD17596, CMNPD22144, CMNPD25994, and CMNPD17598) based on their better docking score and interaction profile, for in-depth analysis through re-docking procedures against Marburg VP35 (Table 1). The re-docking scores were as follows: CMNPD17596 at −6.22 kcal/mol, CMNPD22144 at −5.79 kcal/mol, CMNPD25994 at −5.66 kcal/mol, and CMNPD17598 at −5.56 kcal/mol. These results indicated a higher affinity of the selected compounds for the VP35 protein compared to the reference compound.

### 2.2. Molecular Interaction Analysis of the Docked Complexes

**Figure 1:**
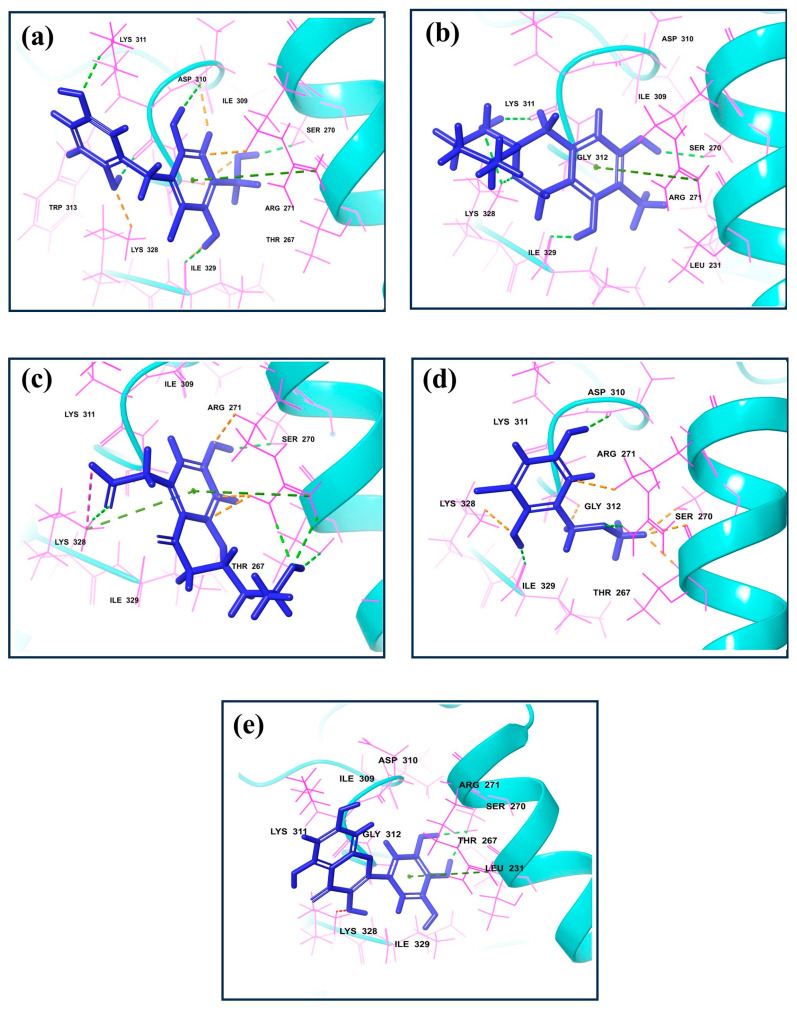
3D interaction for the dsRNA binding domain with natural compounds, i.e., (**a**) CMNPD17596, (**b**) CMNPD22144, (**c**) CMNPD25994, (**d**) CMNPD17598, and (**e**) Control resulting from molecular docking.

**Figure 2:**
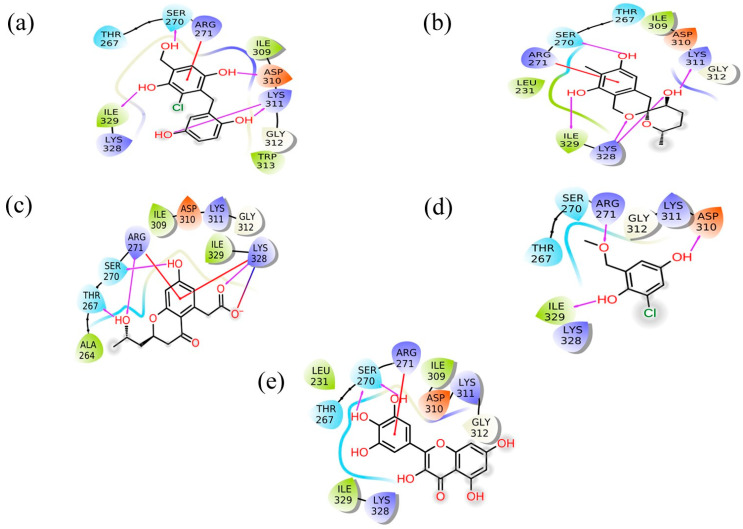
2D interaction plot for the Marburg virus VP35 and marine fungi compounds generated by free academic maestro [25]: (**a**) CMNPD17596, (**b**) CMNPD22144, (**c**) CMNPD25994, (**d**) CMNPD17598, and (**e**) Control/Myricetin.

The molecular interaction profiles of these compounds with VP35 revealed distinct binding patterns, contributing to their docking scores (Figure 1 and Figure 2).

CMNPD17596 exhibited a total of five hydrogen bonds and one Pi–cation interaction (Figure 2a). The hydrogen bonds were formed with SER270, ASP310, two with Lys311, and ILE329. The Pi–cation interaction was observed with ARG271, indicating a robust interaction profile (Figure 2b). CMNPD22144 also formed five hydrogen bonds and one Pi–cation interaction (Figure 2b). The hydrogen bonds were with SER270, ASP329, Lys328 (two bonds), and Lys311. The Pi–cation interaction was similar to CMNPD17596 (Figure 2a), occurring with ARG271. CMNPD25994 showed a slightly different pattern with four hydrogen bonds, one Pi–cation interaction, and a salt bridge (Figure 2c). The hydrogen bonds were with Thr267, SER270, Arg271, and Lys328. The Pi–cation interaction and salt bridge both involved Lys328 and Arg271, suggesting a unique binding mode. CMNPD17598 demonstrated three hydrogen bonds, interacting with Arg271, ASP310, and ILE329 (Figure 2d). This simpler interaction profile suggested a different mode of binding compared to the other compounds. Myricetin formed two hydrogen bonds with SER270 and a Pi–cation interaction with Arg271, indicating a less extensive interaction network compared to the screened compounds (Figure 2e).

Besides these specific interactions, all compounds and the reference showed various hydrophobic and polar contacts with other critical binding site residues of the Marburg VP35, contributing to their overall binding affinity. Comparing the screened compounds with myricetin, it was evident that the newly identified compounds displayed a more extensive network of molecular interactions with VP35. This was particularly notable in the number and variety of hydrogen bonds and Pi–cation interactions. All these compounds with the higher docking score showed the most extensive interaction profiles, which could be correlated with their better binding affinity. All the docked complexes were superimposed along with the control. The superimposition showed that all the molecules were bound in a similar pattern at the binding pocket of the target protein (Figure S1).

### 2.3. Molecular Dynamics Simulation and Trajectory Analysis

We performed a 200 ns Molecular Dynamics (MD) simulation to examine the flexibility and stability of the top five hit complexes. We monitored the Root Mean Square Deviation (RMSD) of the protein and ligand as well as the Root Mean Square Fluctuation (RMSF) of the protein during this simulation. By measuring the protein–ligand complexes throughout time, these data let us understand how their structural variations and changes occurred. In addition, we examined the formation of hydrogen bonds along the 200 ns trajectories. Understanding the interactions between the protein and ligand inside these complexes was made possible thanks in a significant way to our work. The intensity and nature of these interactions were clarified by the hydrogen bond analysis, which also provided important details regarding the binding stability and possible mechanistic insights.

#### 2.3.1. RMSD and RMSF Analysis

**Figure 3:**
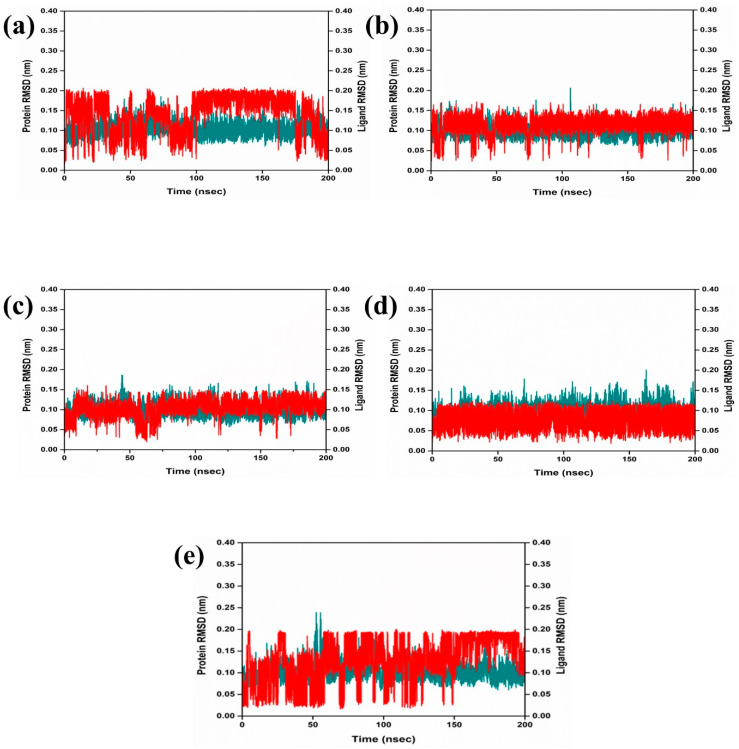
RMSD plot for the Marburg virus VP35 and marine fungi compounds: (**a**) CMNPD17596, (**b**) CMNPD22144, (**c**) CMNPD25994, (**d**) CMNPD17598, and (**e**) Myricetin.

**Figure 4:**
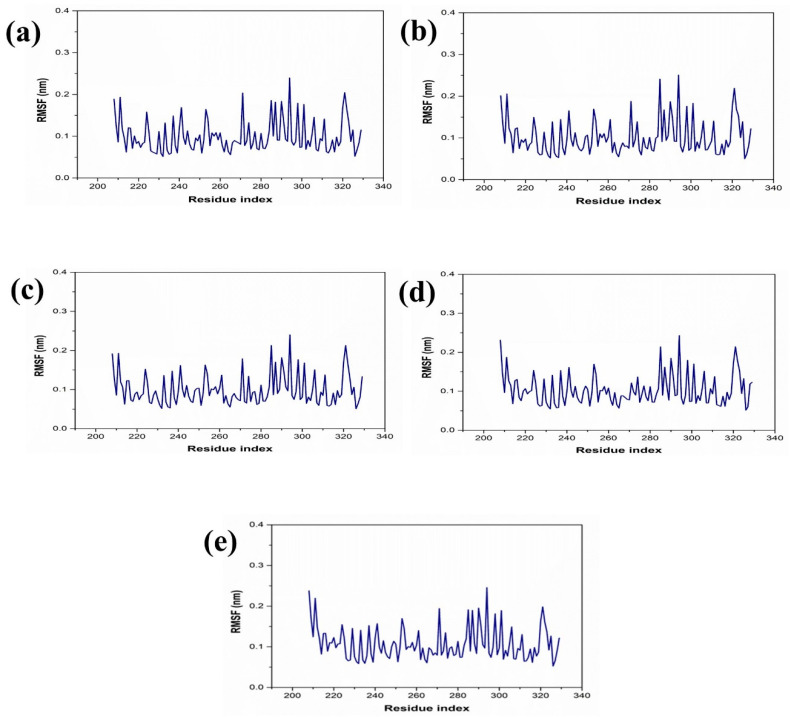
RMSF plot for the Marburg virus VP35 and marine fungi compounds: (**a**) CMNPD17596, (**b**) CMNPD22144, (**c**) CMNPD25994, (**d**) CMNPD17598, and (**e**) Myricetin.

The dynamic behavior and structural characteristics of four naturally occurring chemicals, when bound to the Marburg virus RNA protein, were investigated using a molecular dynamics (MD) simulation. Important insights into the stability and interactions of the protein–ligand complexes as well as the conformational changes taking place within the system were obtained from the simulation. During the 200 ns simulation, the stability of the protein–ligand complexes was evaluated using the root mean square deviation (RMSD) technique. During the examination of RMSD from the simulation trajectory of each complex, it was shown that the VP35-RBD protein in all the selected natural compounds docked complex and reference complex displayed dynamic stability (<0.20 nm) (Figure 3). Also, the RMSD analysis of the CMNPD17596 ligand molecule displayed stable RMSD (<0.15 nm), indicating that the ligand shows substantial stability throughout the simulation while bound to the target protein (Figure 3a). Similarly, the CMNPD22144 natural compound also exhibited a stable RMSD (≤0.1 nm) between a range of 0 to 200 ns time (Figure 3b). Likewise, the CMNPD25994 natural compound showed maximum stability (<0.1 nm) from the start to 200 ns time, with insignificant fluctuations during the RMSD analysis of the simulation trajectory (Figure 3c). The CMNPD17598 natural compound also demonstrated maximum stability (<0.1 nm) until the end of the simulation, indicating significant dynamic stability during their binding to the RNA protein (Figure 3d). The reference molecule showed an RMSD value of <0.2 nm until the 200 ns simulation time, which remained stable (Figure 3e). The RMSD results analysis indicated that all the compounds were stable and may inhibit the target protein when compared to the reference molecule (Figure 4). RMSF is calculated by determining the average deviation of atomic positions from their mean positions throughout the simulation. Higher RMSF values indicate greater flexibility and fluctuation, while lower RMSF values suggest more rigidity and stability. The RMSF value of the VP35-RBD was (<0.2 nm) with a minimum number of residual fluctuations (Figure 4). This states that the VP35-RBD remained stable through the stimulation, and this also supports the protein RMSD analysis of the protein–ligand complexes.

#### 2.3.2. Number of Hydrogen Bond Analysis

**Figure 5:**
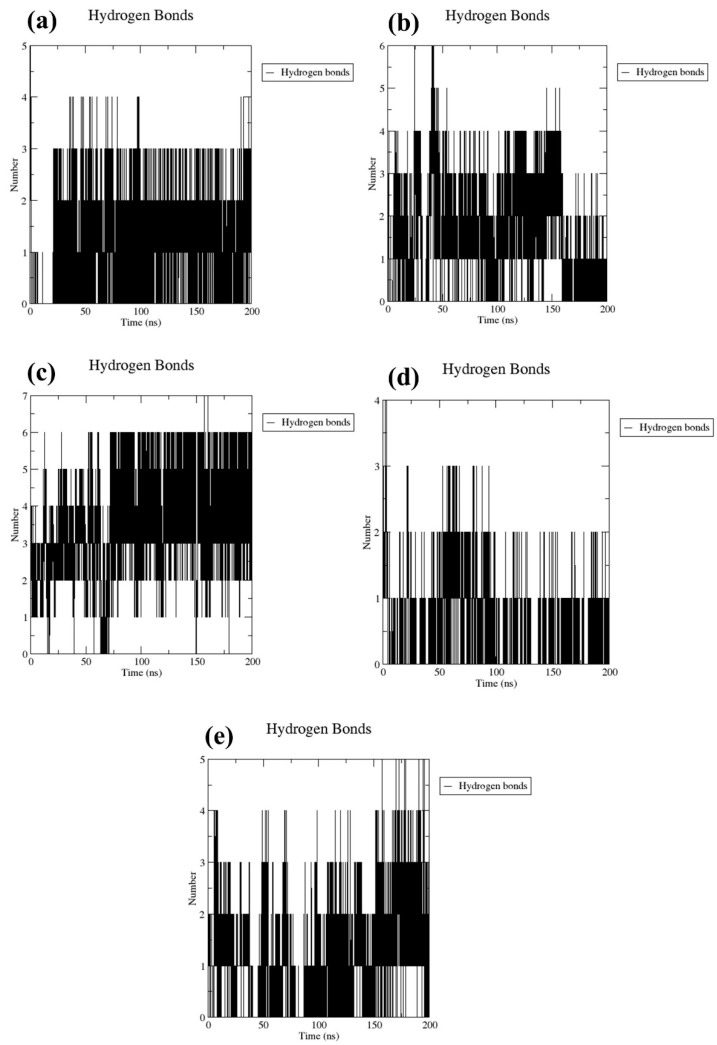
Plots of the number of hydrogen bonds for the Marburg virus VP35 and marine fungi compounds: (**a**) CMNPD17596, (**b**) CMNPD22144, (**c**) CMNPD25994, (**d**) CMNPD17598, and (**e**) Myricetin.

The stability of docked molecular complexes can be inferred from the quantity of hydrogen bonds they form. In this study, both the tested compounds and a control group consistently formed hydrogen bonds, ranging from a minimum of one to a maximum of seven, throughout the entire simulation duration (Figure 5). This included bond pairs within a distance of 0.35 nanometers (3.5 Ångströms), indicating the frequency of interactions within the predefined threshold between the protein and ligand. Specifically, CMNPD17596 (Figure 5a) and CMNPD17598 (Figure 5d) each established up to four interaction points in their respective docked configurations during the simulation process. Furthermore, in most simulation frames, CMNPD22144 exhibited three to six interaction points, denoting a comparable level of conformational stability (Figure 5b). This consistency is believed to play a crucial role in enhancing the binding strength over the course of the simulation. Notably, CMNPD25994 (Figure 5c) demonstrated the formation of the most hydrogen bonds (seven) and the least (three), in contrast to the control complex (Figure 5e), which formed a maximum of five hydrogen bonds with the target protein. Under these circumstances, the docked ligands have the potential to alter the flexibility of the protein and decrease the enzymatic activity. These observations were also supported by the computed RMSD and RMSF of the protein.

### 2.4. Principal Component Analysis

**Figure 6:**
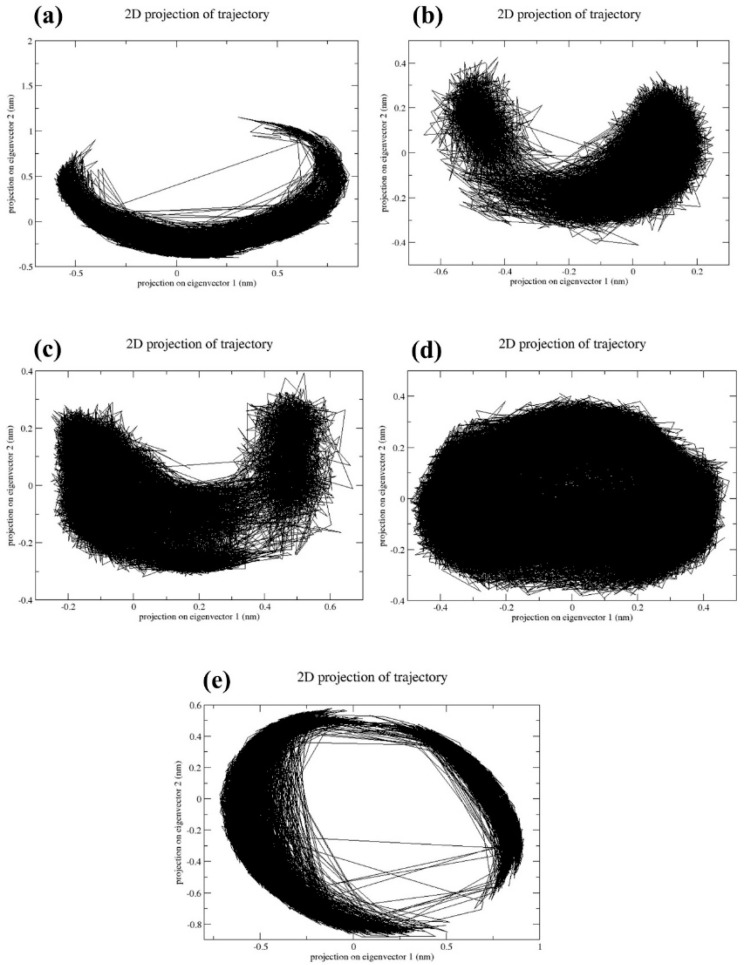
Principal component analysis of the two primary components (PC1 and PC1) for the complexes of (**a**) CMNPD17596, (**b**) CMNPD22144, (**c**) CMNPD25994, (**d**) CMNPD17598, and (**e**) Myricetin.

The reduction in dimensions is crucial for understanding the quality and output of the multidimensional data. Principal component analysis (PCA) is a major technique used to reduce the dimensions of multidimensional data. In Figure 6, it was observed that a CMNPD17596 complex exhibited a less dense scatter plot (Figure 6a). This indicates that the complex has undergone conformational changes during the simulation process. However, the CMNPD22144 (Figure 6b), CMNPD25994 (Figure 6c), and CMNPD17598 (Figure 6d) docked complexes showed dense scatter plots, indicating that the complex has shown a very minimum amount of confirmational changes during the simulation time. This states that these three complexes are comparatively much more stable than CMNPD17596 (Figure 6a). The reference complex myricetin exhibited a less dense plot, which states that the reference complex has also undergone conformational changes (Figure 6e). Based on the PCA results obtained from the scatter plot, the CMNPD22144 (Figure 6b), CMNPD25994 (Figure 6c), and CMNPD17598 complexes (Figure 6d) had much more dynamic stability than the reference complex (Figure 6e), due to the presence of fewer conformational changes.

### 2.5. Free Energy Landscape

**Figure 7:**
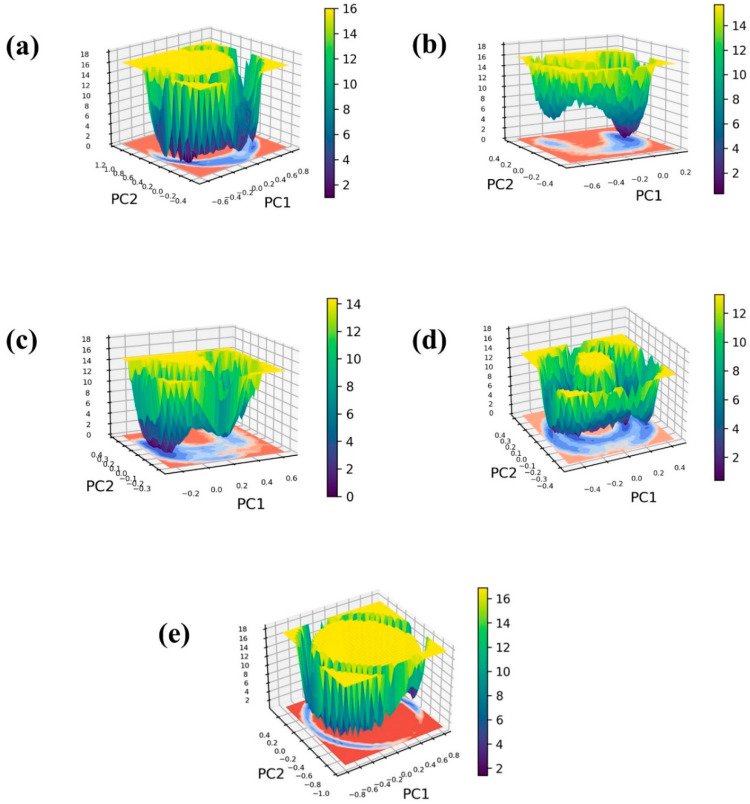
Free energy landscape of the Marburg virus VP35 and marine fungi compounds: (**a**) CMNPD17596, (**b**) CMNPD22144, (**c**) CMNPD25994, (**d**) CMNPD17598, and (**e**) Myricetin.

To construct the 3D free energy landscape, we generated graphs illustrating the first principal component (PC1) and the second principal component (PC2) obtained from the PCA analysis. Figure 7 displays the resultant free energy landscapes for both the ligand-bound complexes and a control group, offering a visual representation of the energy implications associated with conformational shifts. These graphical depictions serve as valuable tools for understanding the dynamic alterations in conformation throughout the simulation, ultimately revealing the emergence of a low-energy structure resembling a narrow, funnel-like shape. Furthermore, the energy transition bar in Figure 7 reveals dark blue regions, indicating the presence of localized energy minima. These minima suggest instances during the simulation where the protein structures reached their lowest energy states. Notably, these complexes exhibit deep basins, signifying the attainment of structures with minimal energy. Within the framework of a three-dimensional free energy landscape, the terms “narrow basin” and “wide basin” categorize distinct types of energy minima or states that molecules can inhabit.

In Figure 7, the complexes CMNPD22144 (Figure 7b) and CMNPD25994 (Figure 7c) are depicted with narrow basins, indicating highly stable states characterized by infrequent energy transitions. In contrast, CMNPD17596 (Figure 7a), CMNPD17598 (Figure 7d), and the reference complex display relatively wide basins, suggesting a spectrum of accessible states with more frequent energy transitions among them. Examining structures corresponding to minimum, intermediate, and maximum energy levels provide insights into the interactions and conformational changes within these complexes. This observed behavior points to a binding arrangement characterized by stability. Notably, the minimum energy state, serving as the reference point, consistently registers at 0 kJ/mol for all complexes. In contrast, the relative maximum energy state falls within the range of 12 kJ/mol to 16 kJ/mol, offering a quantitative measure of the energy landscape across these molecular systems. Herein, all the selected protein–ligand complexes exhibited minimum Gibbs energy between the energy of 0 to 2 KJ/mol. Also, the reference complex shows the minimum Gibbs energy between 0 to 2 KJ/mol. Even though the minimum energy value of the selected complex and control are the same, the exploration of the free energy landscape for CMNPD17596 (Figure 7a), CMNPD17598 (Figure 7d), and the reference molecule myricetin (Figure 7e) reveals the presence of multiple conformers. This observation suggests a potential reduction in the molecule’s stability. In the case of Molecule CMNPD22144 (Figure 7b) and CMNPD25994 (Figure 7c), the free energy landscape showcases a predominant global minimum, coupled with a secondary local minimum possessing a marginally reduced Gibbs free energy. This suggests the molecule may have an alternate conformation that is slightly less stable.

## 3. Discussion

In this study, we have undertaken a computational drug discovery approach, integrating virtual screening with Lipinski’s filter, re-docking, molecular dynamics (MD) simulation, and principal component analysis (PCA), to identify potential MBV VP3-RBD inhibitors from a Marine Fungi Compounds library. Our findings have presented novel insights into the binding affinities and dynamic behaviors of selected compounds, offering a significant contribution to the field of antiviral drug discovery.

The initial virtual screening process was crucial in filtering out candidates with promising drug-like properties, leading to the identification of four compounds with high docking scores. This step is essential in computational drug discovery, as seen in previous studies where initial screenings have successfully narrowed down potential candidates from vast libraries [18,26,27]. The re-docking scores of our selected compounds were higher than that of the reference compound, myricetin, indicating a stronger affinity for the Marburg VP35 protein. This is in line with previous reports where re-docking was used to validate and refine the virtual screening findings [28,29,30].

Molecular contact analysis results revealed diverse binding patterns in each protein–ligand complex, with varying numbers of hydrogen bonds and Pi–cation interactions. Such comprehensive interaction profiles are essential for comprehending the binding mechanisms, as shown in earlier research, wherein Pi interactions and hydrogen bonds were critical for the stability of ligand–protein complexes. Our results advance this understanding by showing how these interactions affect docking scores and the binding efficacy of marine fungi compounds.

The MD simulation and trajectory analysis provided a deeper understanding of the protein–ligand complexes’ flexibility, stability, and interaction dynamics. The observation of stable RMSD values throughout 200 ns MD simulations for all compounds is consistent with other findings, which have underscored the significance of MD simulations in forecasting the dynamic behavior of identified compounds [31,32,33]. RMSF results further supported the stability of the complexes, adding to the understanding of their local conformational behaviors throughout the complete simulation.

The importance of hydrogen bond analysis was also highlighted throughout the simulation duration. The hydrogen bond analysis is a crucial aspect of understanding the stability and binding strength of identified molecules, as also seen in earlier studies [34,35,36,37], where the number of hydrogen bonds is linked to the stability of the protein–ligand complexes.

The PCA results provided valuable insights into the conformational changes and dynamic stability of the complexes. Our findings of minimal conformational changes in most complexes, indicating higher stability, align with the results of similar studies [18,28]. This emphasizes how important PCA is to the drug development process in order to comprehend multidimensional data and the behavior of potential drugs.

Lastly, the free energy landscape analysis offered a visual representation of the energy states and transitions of the complexes. Our observations of narrow basins for certain complexes, indicative of stable states, and wide basins for others suggest varying degrees of stability and conformational flexibility. This aspect of our study adds to the growing body of evidence, as seen in the work of Rabaan and his co-workers [28], that free energy landscapes are critical for understanding the energetics and stability of molecular interactions in drug discovery.

Building upon these findings, our study significantly contributes to the expanding landscape of antiviral research, particularly in the context of MBV inhibitors. The unique properties of compounds from the marine fungi compound library, as revealed in our study, provide a new perspective on potential antiviral agents. This is especially relevant considering the limited number of studies focusing on marine-derived compounds for MBV inhibition, highlighting the novelty and potential impact of our work. Comparatively, our results demonstrate advancements over existing studies by showcasing not only the binding affinities but also the dynamic stability of these compounds within the VP35 protein environment [16,17,18,28]. This comprehensive analysis bridges the gap between static structural insights and dynamic molecular behavior, offering a more holistic view of potential drug efficacy.

Overall, our study has successfully integrated various computational approaches to identify potential inhibitors of the MBV VP35-RBD from a Marine Fungi Compounds library. By comparing our findings with previous studies, we have highlighted the consistency and novelty of our approach in the broader context of computational drug discovery. Our results provide a foundation for further experimental validation and optimization of these compounds as potential therapeutic agents against the MBV.

## 4. Methodology

### 4.1. Data Collection, Virtual Screening, and Re-Docking

Embarking on a journey to combat the Marburg virus, we employed a computational approach to identify inhibitors targeting the VP35 protein. Our initial step involved the downloading of a specialized compound library, sourced from the Comprehensive Marine Natural Products Database (CMNPD) [38]. The CMNPD is an extensive repository of information on natural compounds derived from marine sources. It serves as a valuable resource for researchers in pharmacology, chemistry, and marine biology, offering detailed data on the chemical structure, biological activity, and potential applications of these compounds. The CMNPD facilitates the exploration of marine biodiversity for novel drug discovery and provides insights into the medicinal potential of marine life. Its extensive catalog aids in advancing the field of marine biotechnology and natural product research. Our target protein was the VP35-RBD protein, for which we obtained structural data from the Protein Data Bank, using the specific PDB ID: 4GHA [39]. In this structure, VP35-RBD protein was bound in complex with 12-bp dsRNA at 2.50 Å resolution, which was crucial for accurate molecular modeling experiments.

The initial phase involved preparing this protein for computational experiments. We used the DockPrep tool in the Chimera software suite version 1.17.3, a critical process that involved refining the protein structure to make it suitable for docking studies [40]. This step included removing water molecules, adding hydrogen atoms, and ensuring the protein’s structure was ready for interaction with potential inhibitors.

Our next step was to screen the marine fungi compound library. We conducted this virtual screening using the MTiOpenScreen server, which allowed us to sift through numerous compounds efficiently [22]. An important aspect of this screening was applying Lipinski’s Rule of Five, a set of criteria ensuring that the compounds we selected had properties consistent with good drug candidates, like being absorbable and effectively reaching their target in the body. The CASTp 3.0 server was used to predict the binding pocket and the grid parameters were selected based on predicted binding pocket residues [41].

CASTp 3.0 is well-known for its high accuracy in binding site prediction and aligns well with our molecular docking software. From this screening, we identified four compounds that showed potential as VP35 inhibitors. We included a known reference molecule in our study for comparison. To confirm the initial findings, we performed a re-docking process using the AutoDock Vina plugin in Chimera [42,43]. This step was about making sure that these compounds could indeed bind effectively to the VP35 protein. We prepared the protein and the selected compounds using the DockPrep tool, setting the stage for the docking process.

A critical aspect of the docking was defining the precise location where we expected the compounds to bind to the protein. We set the grid center coordinates to 2.23, 24.56, and 36.36, with a grid size of 20 Å in each dimension. This careful positioning ensured that we could observe how each compound interacted with the protein’s active site. The 2D and 3D interaction diagrams were plotted in free academic Schrodinger maestro to explain the molecular interaction analysis between each protein and ligand complexes [25].

### 4.2. Molecular Dynamics Simulation and Trajectory Analysis

Molecular Dynamics (MD) Simulations were used to assess the efficacy of Marine Fungi Compounds as inhibitors for Marburg VP35. Employing GROMACS-2023.2 software, integrated with the CHARMM Force Field, we conducted a detailed analysis on four selected complexes and one reference compound [44,45].

For each complex, the Marine Fungi Compound was docked with Marburg VP35. These complexes were then positioned in a cubic simulation box, filled with TIP3P water molecules. The minimum separation between the complex and the box boundary was set at 10 Å. An ionic balance was achieved by adding Na+ or Cl- ions, ensuring a physiologically relevant environment. Prior to the MD simulations, each system underwent energy minimization. This step, performed using the steepest descent algorithm, helped in stabilizing the complexes and removing potential energy conflicts. The MD simulations were carried out following the CHARMM Force Field guidelines [45]. A 2-fs time step was selected, and each simulation spanned over a period of 200 nanoseconds. The Nose–Hoover thermostat [46] maintained a consistent temperature of 300 K, and the Parrinello–Rahman barostat [47,48] was employed to keep the pressure steady at 1 atm. After achieving equilibrium, we initiated the production run of the simulations. This phase was crucial to observing the dynamic interactions between the Marine Fungi Compounds and Marburg VP35. The stability and conformational changes in the complexes were meticulously monitored. Post-simulation, we conducted an extensive trajectory analysis. This included calculating the root-mean-square deviation (RMSD) and root-mean-square fluctuation (RMSF) and monitoring the hydrogen bonding patterns. These analyses were instrumental in understanding the binding dynamics and stability of the complexes over the simulation timeframe.

### 4.3. PCA-Based Free Energy Landscape

The final phase of our methodology involved conducting principal component analysis (PCA) based on free energy landscape analysis. For each of the four complexes, along with one reference compound, the principal component analysis (PCA) was utilized to decompose these trajectories into principal components, effectively reducing the dimensionality of the motion data. This process highlighted the dominant motions within the protein–ligand interactions [49]. We then mapped these principal components onto a free energy landscape using the gmx sham module and Matplotlib [50]. This mapping revealed the various conformations adopted by the complexes, highlighting the most energetically favorable states. By comparing these states across the four complexes and the reference, we could infer the relative binding affinities and stability of the marine fungi-derived compounds.

This PCA-based approach provided a detailed understanding of the dynamic behavior and energetic profiles of the potential Marburg VP35 inhibitors, guiding our selection of potential therapeutic candidates.

## 5. Conclusions

This study represents a significant finding to combat the MBV through a computational drug discovery approach. By employing a widely recognized combination of virtual screening, re-docking, principal component analysis, molecular dynamics simulations, and free energy landscape, we were able to effectively pinpoint putative inhibitors (CMNPD17596, CMNPD22144, CMNPD25994, and CMNPD17598) from a Marine Fungi Compounds collection. Comparing the compounds chosen for in-depth analysis to the reference compound, the former showed a greater affinity for the MBV VP35-RBD. The molecular interaction analysis showed complex binding patterns for every drug and suggested a strong interaction with the viral protein. The MD simulations further highlighted these compounds’ potential as effective therapeutic agents by demonstrating their stability and dynamic behavior. Furthermore, a better comprehension of the stability and conformational changes in the protein–ligand complexes was made possible by the PCA data. The energy profiles of these complexes were revealed by the free energy landscape analysis, which provided information about their stability in different scenarios. This study adds to the increasing arsenal against the MBV and emphasizes the usefulness of computational approaches in drug discovery. The promising findings in this study pave the way for additional experimental validation and investigation, which could result in the creation of potent new treatments for this deadly virus.

## Figures and Tables

**Table 1:** Compound ID in CMNPD database, Compound name, and their marine fungi source species name.

Compound ID	Compound Name	Compound Source Species Name
CMNPD17596	Terrestrol G	*Penicillium solitum*, [24]
CMNPD22144	Peneciraistin A	*Penicillium raistrickii*, [24]
CMNPD25994	Corynechromone F	*Corynespora cassiicola*, [24]
CMNPD17598	2-chloro-6-(methoxymethyl)benzene-1,4-diol	*Penicillium solitum*, [24]

## Data Availability

The datasets used and analyzed during the current study are available from the corresponding author upon reasonable request.

## Supplementary Materials

The following supporting information can be downloaded at: https://www.mdpi.com/article/10.3390/md22010034/s1, Table S1. Virtual screening results of top 42 marine fungi compounds; Figure S1: Superimposition of all the four docked complexes along with the control molecule.
