# Supplementary material for: Structural and Dynamical Basis of VP35-RBD Inhibition by Marine Fungi Compounds to Combat Marburg Virus Infection
Source: Mar Drugs. 2024 Jan 3;22(1):34. doi: 10.3390/md22010034 (PMC10820117; doi:10.3390/md22010034)
Supplement: Supplementary file 1 [file marinedrugs-22-00034-s001.zip › marinedrugs-2770520-SI.pdf]

Table S1. Virtual screening results of top 42 marine fungi compounds

Compound ID	docking score
CMNPD17596	-6.222017794
CMNPD22144	-5.797751052
CMNPD25994	-5.666367572
CMNPD17598	-5.560868403
CMNPD26195	-5.523765329
CMNPD27388	-5.502109939
CMNPD28767	-5.479385846
CMNPD27648	-5.362954408
CMNPD27401	-5.340855903
CMNPD27626	-5.327845723
CMNPD27645	-5.278221828
CMNPD15725	-5.243814154
CMNPD12445	-5.214903359
CMNPD7998	-5.20042287
CMNPD23468	-5.194411436
CMNPD25995	-5.177741105
CMNPD29213	-5.151133443
CMNPD30500	-5.149066556
CMNPD25997	-5.147268401
CMNPD22277	-5.142960793
CMNPD28915	-5.128329015
CMNPD30608	-5.123334927
CMNPD21013	-5.122316859
CMNPD30598	-5.116652716
CMNPD26374	-5.11166371
CMNPD30815	-5.108272852
CMNPD27482	-5.107616347
CMNPD30813	-5.090706929
CMNPD24749	-5.085449987
CMNPD29152	-5.083457305
CMNPD22074	-5.077837488
CMNPD27538	-5.076164635
CMNPD25996	-5.051254963
CMNPD27623	-5.049201072
CMNPD30950	-5.036260929
CMNPD17597	-5.035130563
CMNPD23567	-5.030194443
CMNPD17592	-5.025864034
CMNPD23544	-5.011872256
CMNPD29168	-5.009317178
CMNPD30609	-5.009166743
CMNPD9455	-5.001829611

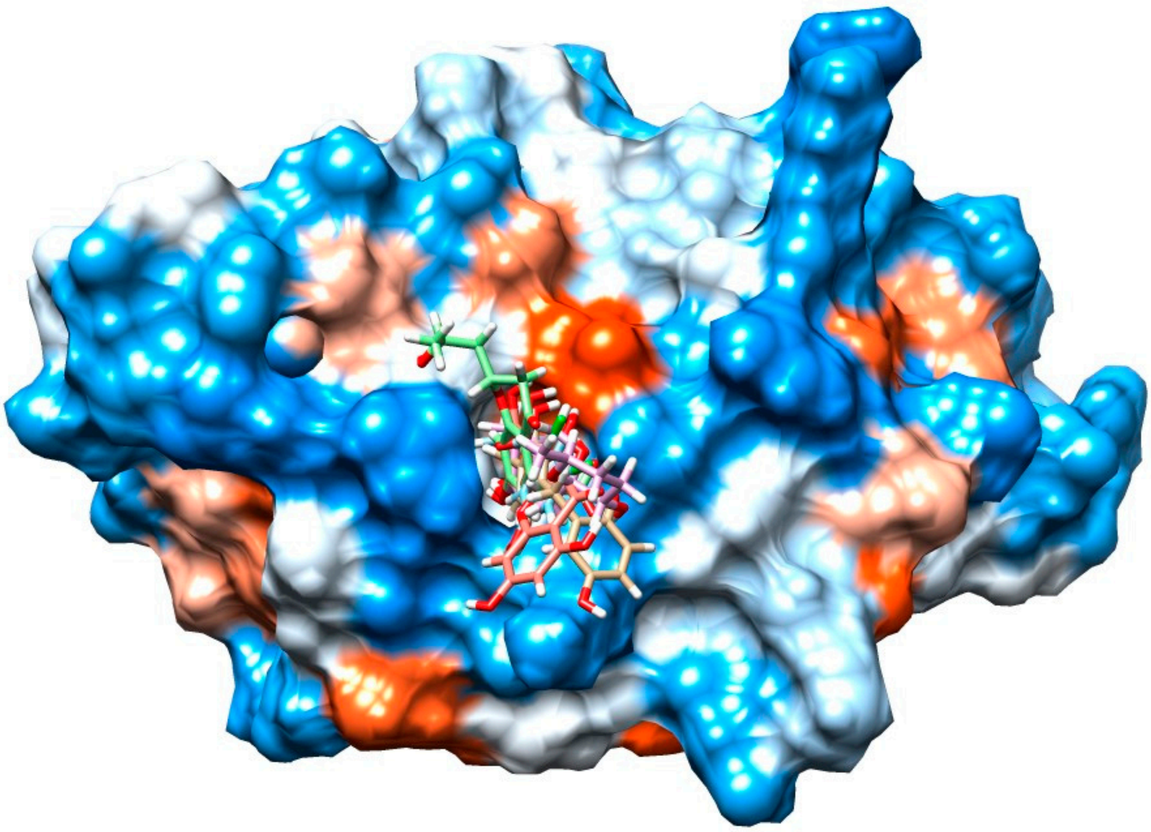

Figure S1: Superimposition of all the four docked complexes along with the control molecule.
